# Two-Step Thermochemical Cellulose Hydrolysis With Partial Neutralization for Glucose Production

**DOI:** 10.3389/fchem.2018.00117

**Published:** 2018-04-24

**Authors:** James Kong-Win Chang, Xavier Duret, Véronique Berberi, Hassan Zahedi-Niaki, Jean-Michel Lavoie

**Affiliations:** ^1^Industrial Research Chair on Cellulosic Ethanol and Biocommodities, Department of Chemical and Biotechnological Engineering, Université de Sherbrooke, Sherbrooke, QC, Canada; ^2^CRB Innovations Inc., Sherbrooke, QC, Canada

**Keywords:** cellulose hydrolysis, sulfuric acid, glucose, partial neutralization, triticale

## Abstract

Cellulose hydrolysis processes using concentrated acid usually involve two steps in order to obtain high glucose yields. The first step (pre-treatment) decrystallizes cellulose while the second step (post-hydrolysis) converts the amorphous cellulose to glucose. The two-step process developed by the Industrial Research Chair on Cellulosic Ethanol and Biocommodities and its industrial partner CRB Innovations Inc., includes an intermediate partial neutralization step, whose purpose is to decrease the amount of dilution water to be added for post-hydrolysis thus minimizing handling costs. In this work, the effect of several operating parameters on the glucose yield of this process was investigated using triticale cellulose and the best conditions yielding fermentable glucose (close to 100%) were determined. These conditions involve pre-treating cellulose at 30°C using 72 wt% H_2_SO_4_ with a H_2_SO_4_/dry cellulose mass ratio of 36 over 2 h, followed by a partial neutralization using 20 wt% NaOH at an H^+^/OH^−^ molar ratio of 2.3–2.5 and a post-hydrolysis at 121°C for 10 min.

**HIGHLIGHTS**

- Influence of operating parameters on the glucose yield have been investigated.
- Conditions for producing cellulosic glucose with yields close to 100% have been identified.

## Introduction

During the last two decades, there has been tremendous interest in biofuels, as a result of ever-increasing worldwide energy demand and over-dependence on oil (Lee and Lavoie, 2013). The major drop in oil barrel price from US$ 107.26 in 2014 to US$ 29.64 in 2016 has negatively impacted the biofuel industry with two major companies in the field filing for bankruptcy (Lavoie, 2016). However, the interest toward increasing the production of biofuels is still very strong. Since renewable fuels have a carbon balance close to neutrality (Lee and Lavoie, 2013), they are essential to mitigate the adverse climate change and global warming effects caused by greenhouse gas emissions from petroleum-derived liquid fuels.

First generation liquid biofuels, produced primarily from food crops such as sugar crops, oil seeds, and cereals, represent a mature commercial technology and market, but face controversies in terms of their sustainability and potential to meet governmental targets. To some extent, these biofuels compete for land and water used for food and fiber production. Moreover, their production and processing costs are often high as compared to fossil fuels and hence they require governmental subsidies to be able to compete with petroleum products. In addition, their greenhouse gas reductions may be impacted from considering land-use change (Sims et al., 2010). Ethanol from corn starch is an example of first generation liquid biofuel and similarly to starch, cellulose is a glucose-based polymer that can be hydrolyzed to liberate monosaccharides, which can then be fermented to ethanol. Cellulose is an attractive raw material since it is readily available from relatively cheap feedstock such as agricultural and forest residues priced between US$ 60 and US$ 80 per ton (Lee and Lavoie, 2013). However, the biggest challenge with cellulose is its recalcitrant structure, making it challenging to be efficiently converted using chemical or biological approaches.

Cellulose is a linear polymer of D-anhydroglucopyranose monomers connected by β-1,4-glycosidic bonds (Zhang et al., 2012) and its degree of polymerization varies typically between 2,000 to 27,000 glucan units, depending on the type of plant (Taherdazeh and Karimi, 2007). It is found as stacks of linear chains with D-cellobiose repeating units and these chains are closely packed, with intramolecular hydrogen bonding within each single glucan chain and intermolecular hydrogen bonding between adjacent chains. The resulting fibrous structure and high degree of crystallinity of cellulose accounts for cellulose recalcitrance (Xiang et al., 2003; Pulidindi et al., 2014). As a first step to overcome this recalcitrance, pre-treatment of biomass is usually performed. When the main aim is to produce ethanol from the cellulosic part, the pre-treatment step also serves the purpose of removing lignin and hemicelluloses, decreasing cellulose crystallinity while increasing the material porosity and freeing cellulose from the tightly-woven lignocellulosic biomass structure (Sun and Cheng, 2002; El-Zawawy et al., 2011).

Acid hydrolysis of cellulose is a classic way to break down cellulose into glucose and can be done using either dilute acid or concentrated acid. The main advantage of cellulose hydrolysis using dilute acid is that the acid does not need to be recovered (Ni et al., 2013) but its several disadvantages include the need of high temperature (at least 180°C) and pressure (around 10 atm) which still provides low glucose yields (Iranmahboob et al., 2002). For example, cellulose hydrolyzed with 0.4 wt% acid at 215°C for 3 min after pre-treatment (hemicelluloses hydrolysis) with 0.7 wt% sulfuric acid at 190°C for 3 min gave a glucose yield of only 50% (Hamelinck et al., 2005). On the other hand, cellulose hydrolysis using concentrated acid takes place at moderate temperature and pressure and results in higher glucose yields (around 90%; Hamelinck et al., 2005), but usually involves a longer reaction time (2–6 h) and it is difficult to economically separate the glucose while recovering the acid.

The concentrated acid hydrolysis of cellulose historically started in 1883 when the method of dissolving and hydrolyzing cotton cellulose with concentrated sulfuric acid was invented. From 1937 to 1948, three processes for concentrated acid hydrolysis of cellulose, namely the Bergius-Rheinau process (Kent, 2013; Amarasekara, 2014), the Peoria process, and the Hokkaido process (Wenzl, 1970; Clausen and Gaddy, 1993) were deployed at large scale. However, they were only successful at times of national crisis, when oil from the Middle East was not readily accessible, and fell out of use after World War II due to poor yields, significant waste streams and large quantities of unmarketable by-products (Arkenol, 1999). Complicated process units, high energy consumption and difficulties to recycle acid were also mentioned as obstacles toward the commercialization of these technologies (Fan et al., 1987). In the mid-1970s, the Peoria process was improved at Purdue University and at the Tennessee Valley Authority (TVA) by recycling dilute acid, and became known as the TVA process. In 1989, Arkenol, an American company, developed a two-stage concentrated acid hydrolysis process using a chromatographic method to separate sugar and acid (Amarasekara, 2014). BlueFire Renewables Inc., established in 2010 to deploy the Arkenol technology (BlueFire Renewables, 2010), was awarded funding from the US Department of Energy and a letter of intent from the Export Import Bank of China was renewed in February 2015 to provide up to 270 million US$ in debt financing for its intended commercial plant in Fulton, Mississippi. Once the financing was completed, the plant would be constructed (BlueFire Renewables, 2015).

CRB Innovations Inc., one of the industrial sponsors of this work, has been working on residual biomass valorization since its creation in 2006. Based on widely published work carried out since the 90's at the University of Sherbrooke, lignocellulosic biomass is first separated into four fractions, namely hemicelluloses, cellulose, lignin, and extractives (if desired) (Lavoie et al., 2011). The cellulosic fraction is then subjected to swelling and decrystallization using concentrated sulfuric acid; the cellulose is “liquefied” in the highly ionic medium. From there on, the oligosaccharides, obtained as a result of the “ionic liquefaction,” are converted into monomeric sugars by a post-hydrolysis step using dilute acid. Variations of this method have the potential of being economically attractive if a minimum acid/cellulose ratio is used for the ionic liquefaction step and a high glucose yield is obtained after post-hydrolysis. Additional requirements include recovering and recycling the used ions, and conditioning the final glucose solution for subsequent fermentation using established microbial systems.

The specific process considered in this study (see **Figure 2**) involves partially neutralizing the acid and decrystallized cellulose mixture by adding aqueous ammonia or sodium hydroxide solution prior to the post-hydrolysis step. Partial neutralization is done in order to reduce the amount of water required to dilute the sulfuric acid for post-hydrolysis (Chornet et al., 2010). After the post-hydrolysis step, different recovery techniques could be used to separate and regenerate acid from the glucose solution obtained. Membrane electrolysis enables the simultaneous separation and regeneration of sulfuric acid and the base from their salt through reactions at the electrodes. Using electrodialysis, sulfuric acid and sodium sulfate or ammonium sulfate can be separated from the glucose solution but an additional technique is needed to separate the sulfuric acid from the salt and regenerate the base. Chromatographic methods could also be used for the separation of ions and glucose and have been extensively reported in literature (Neuman et al., 1987; Foody and Tolan, 2006; Wahnon, 2008; Heinonen and Sainio, 2010; Mahnon, 2010; Heinonen et al., 2012). After separating the ammonium sulfate from the glucose solution, the salt can be subjected to pyrolysis to produce sulfuric acid and ammonium hydroxide (Hansen, 2004). Each of these recovery techniques has its own advantages and disadvantages, and the ultimate best choice depends on many factors, such as the recovery (separation) efficiency and the energy cost associated (Berberi, 2010).

The objectives of this work are to investigate the influence of key parameters of the two-step hydrolysis process with partial neutralization on the glucose yield, and to identify the conditions resulting in the highest glucose yield.

## Materials and methods

### Feedstock

**Figure 1:**
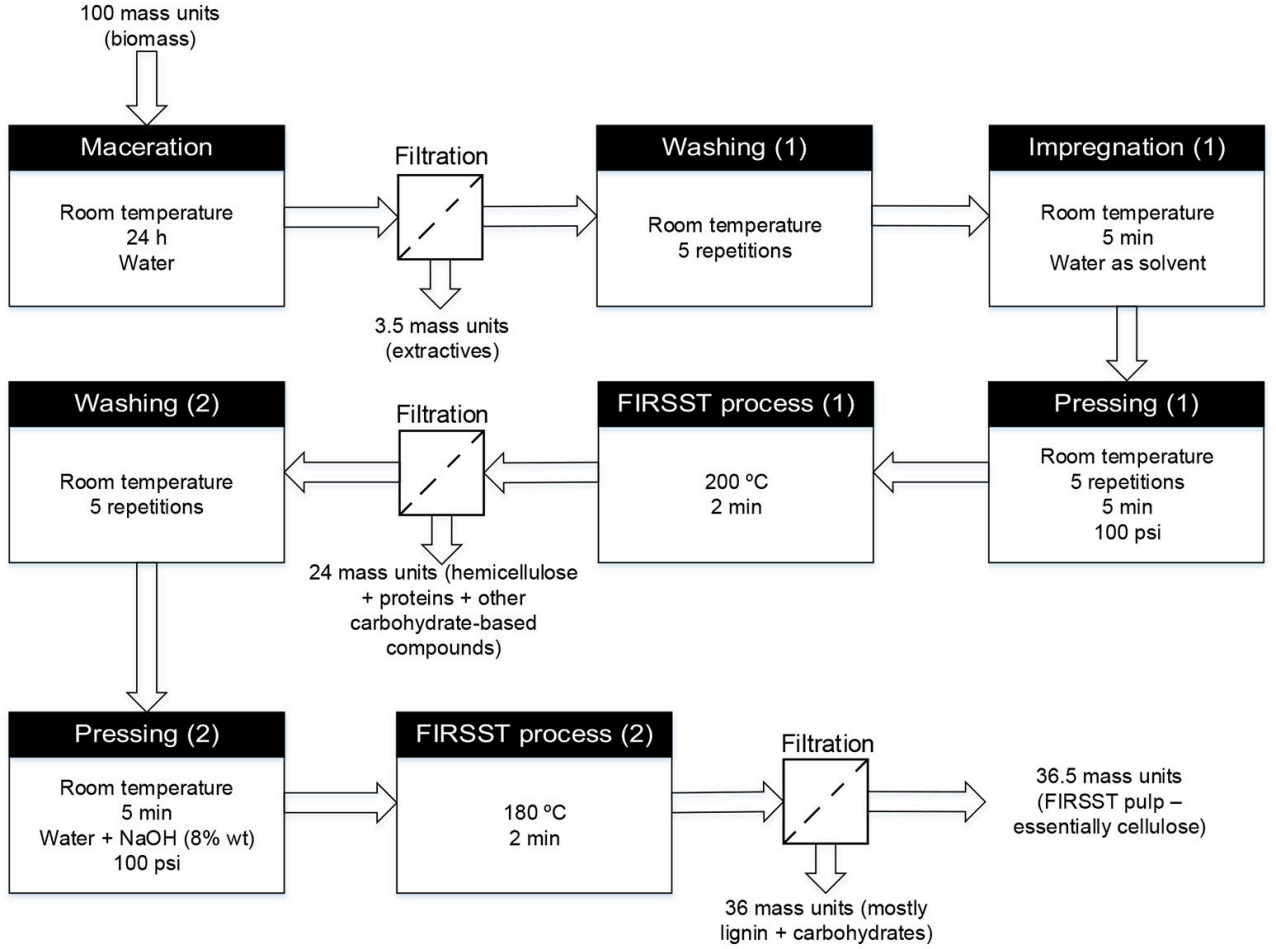
Schematic diagram of the FIRSST method used to separate the constitutive fractions of lignocellulosic biomass (adapted from Lavoie et al., 2011).

The cellulose used as feedstock was obtained from pronghorn spring triticale. The biomass was planted on May 19, 2009 by CÉROM (*Centre de recherche sur les grains*) and CRSAD (*Centre de recherche en sciences animales de Deschambault*), and treated with the Buctril M herbicide on June 14, 2009. The triticale was harvested at maturity on September 16, 2009 and the biomass production yield was 175 kg/m^2^. Given the fibrous texture of the straw (non-edible part of the plant), the latter was mechanically cut to approximately 3 cm-long pieces in order to facilitate the downstream treatment. The triticale straws were fractionated using the FIRSST (Feedstock Impregnation Rapid and Sequential Steam Treatment) method, consisting of two steam treatments (as depicted in Figure 1). The first treatment solubilizes the hemicelluloses fraction whereas the second treatment, carried out using an alkaline catalyst, allowed the solubilization of lignin (Lavoie et al., 2011). The remaining solid fraction consisted mainly of cellulose.

Prior to the first steam treatment, biomass was impregnated with water for 24 h at room temperature. Once the secondary metabolites were dissolved in the water, biomass was filtered and washed five times with water by successive impregnations and pressings. The extracted feedstock was then subjected to a first steam treatment carried out at 200°C for 2 min. The resulting mixture was filtered to recover the hemicelluloses in the solution and the biomass was then washed five times with water. Afterwards, the biomass was impregnated with an 8 wt% sodium hydroxide solution and subjected to a second steam treatment at 180°C for 2 min. The mixture from this second steam treatment was filtered and the solid fraction obtained was used as cellulose feed in this research work.

All reagents used in this work were of ACS grade. These include the 95–98 wt% sulfuric acid used for pre-treatment and the bases (29 wt% aqueous ammonia and 20 wt% sodium hydroxide) used for partial neutralization. Moreover, an aqueous 32.8 wt% sodium hydroxide solution was instead used as catalyst (basic) in the experiments aiming at determining the effect of time, acid concentration and temperature of post-hydrolysis on glucose yield.

### Standard cellulose hydrolysis test (ASTM E1758-01)

The standard procedure for the quantitative determination of carbohydrate in cellulose was carried out following the ASTM International method (E1758-01) where: 4.92 g of 72 wt% sulfuric acid was added to 0.3 g of cellulose for 1 h at 30°C with agitation every 15 min. The solution was then diluted to 4 wt% sulfuric acid by adding 84 mL of water, before being placed in an autoclave at 121°C for 1 h. After 20 min of cooling and neutralization to pH 5–6, the mixture was filtered (0.2 μm) and the glucose concentration was measured by high performance liquid chromatography (HPLC) (ASTM International, 2007).

### Cellulose humidity

Prior to hydrolysis, cellulose humidity was determined first by manually pulling the cellulose apart into small pieces and placing approximately 5 g in an aluminum weighing dish previously dried at 105°C. The aluminum weighing dish and cellulose were put in an oven at 105°C for 24 h and weighed, after cooling in a desiccator. The measured mass difference with respect to the initial mass of humid cellulose was calculated as being the percentage humidity of the cellulose. Each test was performed in triplicate and the mean humidity was calculated and used as reference for the mass balance of the hydrolysis tests.

### Cellulose hydrolysis process

The cellulose hydrolysis process involved seven main parameters and the influence of each parameter on glucose yield was investigated in turn, while keeping the other parameters constant. These parameters and their range of values are given in Table 1.

**Table 1:** Main parameters of the hydrolysis process and range of values investigated.

**Step**	**Parameter**	**Range**
Pre-treatment	Mass ratio of sulfuric acid/dry cellulose (*R*_1_)	12–36
	Mass percentage of sulfuric acid (*C*_1_)	62–82 wt%
	Time (*t*_1_)	0–2 h
Partial neutralization	Acid/base molar ratio (*R*_2_)	1–3
	Concentration of base (*C*_2_)	29 wt% NH_3(aq)_
		20–40 wt% NaOH
Post-hydrolysis	Time (*t*_2_)	0–3 h
	Temperature (*T*_1_)	97–121°C

The effect of mass percentage of sulfuric acid and acid/base molar ratio were investigated at all three values of mass ratio (12, 24, 36) of sulfuric acid/dry cellulose but using the best values of pre-treatment time, concentration of base, post-hydrolysis time, and temperature giving the highest glucose yields. The influence of pre-treatment time on glucose yield was then examined at mass ratio of sulfuric acid/dry cellulose of 36, with the best values found for the remaining parameters_._ Finally, the effect of concentration of base, post-hydrolysis time and temperature on the glucose yield was determined, one after the other, using the most desirable (lowest) value of mass ratio of sulfuric acid/dry cellulose (12) and the most efficient values of the other parameters.

Since partial neutralization step is the innovative aspect of this process, the trials investigating the influence of acid/base molar ratio on the glucose yield were carried out in triplicates. With regards to the other experiments, only the trials with conditions giving the highest glucose yield were carried out in triplicates to confirm the findings. The results for the trials carried out in triplicates are presented with error bars representing the standard deviation in their respective figures.

**Figure 2:**
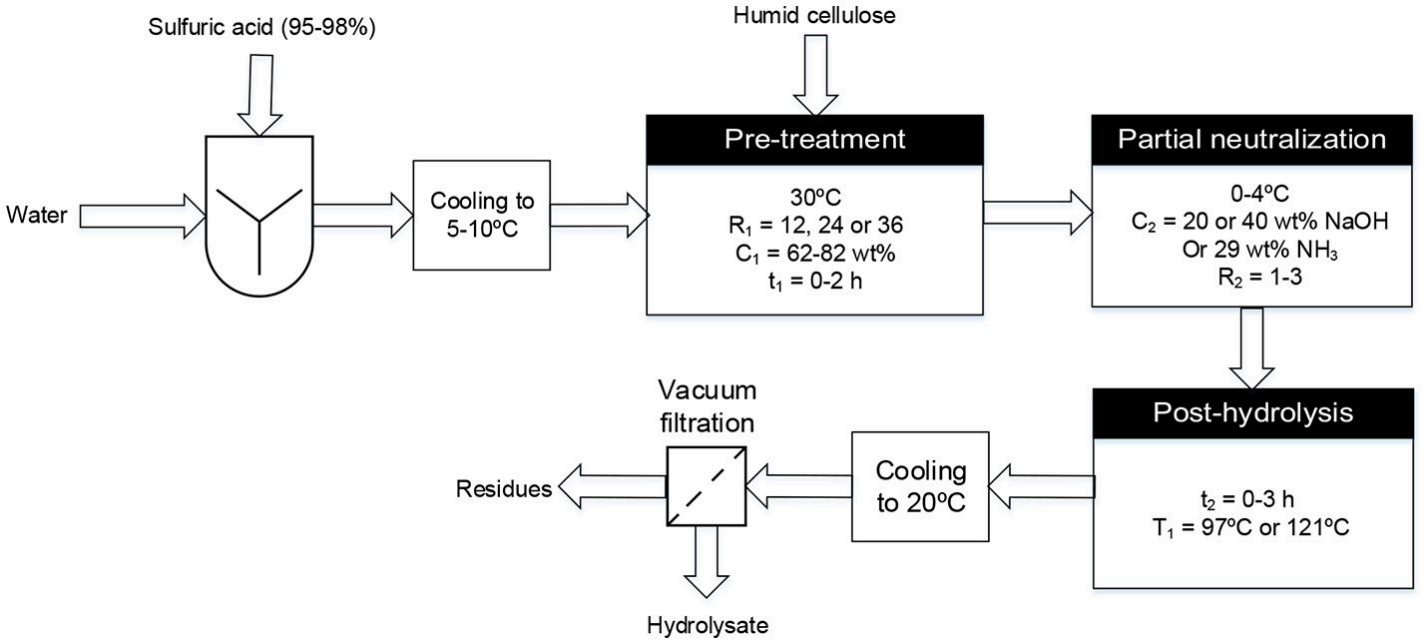
Schematic diagram of the two-step hydrolysis process used to convert triticale cellulose to second generation sugars.

The cellulose hydrolysis protocol itself first involved the preparation of an acid solution by mixing some 95–98 wt% sulfuric acid with water, to get the required mass ratio of sulfuric acid/dry cellulose (*R*_1_) and mass percentage of sulfuric acid (*C*_1_) (when accounting for cellulose humidity) and cooled to 5–10°C in an ice-cold water bath. This acid solution was then added to 5 g of ground cellulose (60–65% humid) in a 250-mL Erlenmeyer flask, while simultaneously agitating with a glass rod. The Erlenmeyer flask was placed in a temperature-regulated bath at 30°C for the desired time (*t*_1_) and its contents stirred using a glass rod at 15 min intervals. At the end of this cellulose pre-treatment process, the Erlenmeyer flask was rapidly cooled by dipping it in a 1 L beaker containing ice-cold water and the appropriate amount of basic solution [NaOH or NH_3(aq)_] added dropwise with agitation, until the desired acid/base molar ratio (*R*_2_) was achieved. This reaction mixture was then post-hydrolyzed using the desired time (*t*_2_) and temperature (*T*_1_). Post-hydrolysis at 121°C was carried out an autoclave while post-hydrolysis at 97°C was performed in a temperature-regulated bath. The Erlenmeyer flask was cooled to 20°C and its contents vacuum-filtered using a 1.5 μm porosity glass microfiber filter (VWR brand) for medium retention particles using a Buchner funnel. The hydrolysate obtained from this step was analyzed to determine its glucose and 5-hydroxymethyl furfural (5-HMF, a glucose degradation product) concentration (see section Determination of Glucose and 5-HMF Concentrations) and to calculate the glucose yield from cellulose hydrolysis. These steps are depicted in Figure 2.

### Determination of glucose and 5-HMF concentrations

Glucose and 5-HMF concentrations were measured with an Agilent brand HPLC equipped with a refractive index detector and a RoA-Organic acid (8%) column (Phenomenex). The column was calibrated with glucose and 5-HMF in a 10–1,000 ppm concentration range. The eluent was a 5 mM aqueous H_2_SO_4_ mixture operated at a flow-rate of 0.6 mL/min and maintained at 60°C. A 30 μL sample was injected for each analysis. Glucose yield from cellulose hydrolysis was calculated as a ratio of the total mass of glucose obtained relative to that obtained by hydrolyzing the same cellulose using the standard ASTM E1758-01 method.

## Results and discussion

The triticale straw used in this work were the same as the one characterized by Beauchet et al. (2013) in their study and were found to contain 12.4 wt% extractives, 31.7 wt% hemicelluloses, 34.0 wt% cellulose, 17.0 wt% lignin, and 4.7 wt% ashes. Moreover, the dual steam treatments enabled recovering 94% of the cellulosic fraction, which had a 60–65% humidity content and consisted of about 7 wt% lignin and 90 wt% glucose (found as glucan chains) on a dry basis.

### Pre-treatment by an aqueous concentrated acid solution

#### Influence of acid concentration

**Figure 3:**
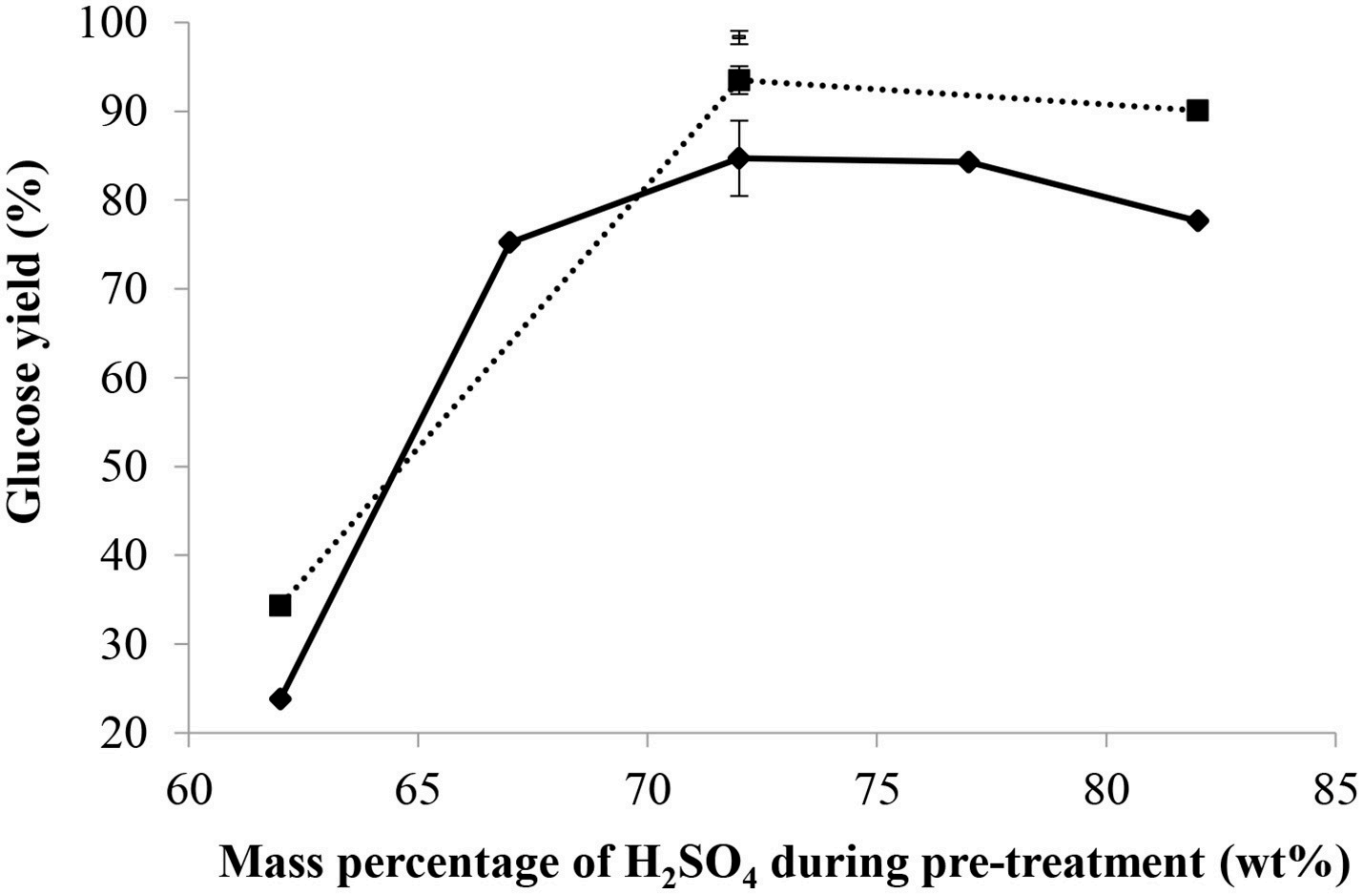
Effect of the mass percentage and quantity of H_2_SO_4_ used during pre-treatment on the glucose yield with mass ratio of H_2_SO_4_/dry cellulose = 12(♦), 24(■), 36(**–**), pre-treatment of 2 h at 30°C, molar ratio of H^+^/OH^−^ = 2.3 for partial neutralization, and 10 min and 121°C for post-hydrolysis.

As shown in Figure 3, with a mass ratio of acid/dry cellulose of 12 and a mass percentage of acid of 62 wt%, the glucose yield was 24% and increased to 85% when the mass percentage of acid reached 72 wt%. At this ratio (12), increasing the mass percentage of acid slightly decreased the glucose yield since the latter was found to be 78% at a mass percentage of acid of 82 wt%. When using a higher mass ratio of sulfuric acid/dry cellulose of 24, the glucose yield rose from 34 to 94% when the mass percentage of acid was increased from 62 to 72 wt%. Again in this situation, increasing the mass percentage of acid to more than 72 wt% did not improve the glucose yield. At a still higher mass ratio of sulfuric acid/dry cellulose of 36 and combined with a H_2_SO_4_ mass percentage of 72 wt%, a glucose yield of 98% was obtained, with a much smaller standard deviation among the triplicates than at lower mass ratios of sulfuric acid/dry cellulose. Seventy-two percentage by weight was therefore found to be the best mass percentage of sulfuric acid, regardless of the mass ratio of H_2_SO_4_/dry cellulose. Moreover, 5-HMF was detected in the hydrolysates but its concentration was below the lower limit of quantification of the HPLC.

Xiang et al. (2003) also found a mass percentage of 72 wt% sulfuric acid to be the optimum for cellulose hydrolysis, although the mass ratio of acid/dry cellulose ratio used in their case was only 19.6 and the highest mass percentage of acid investigated was 72 wt%.

**Figure 4:**
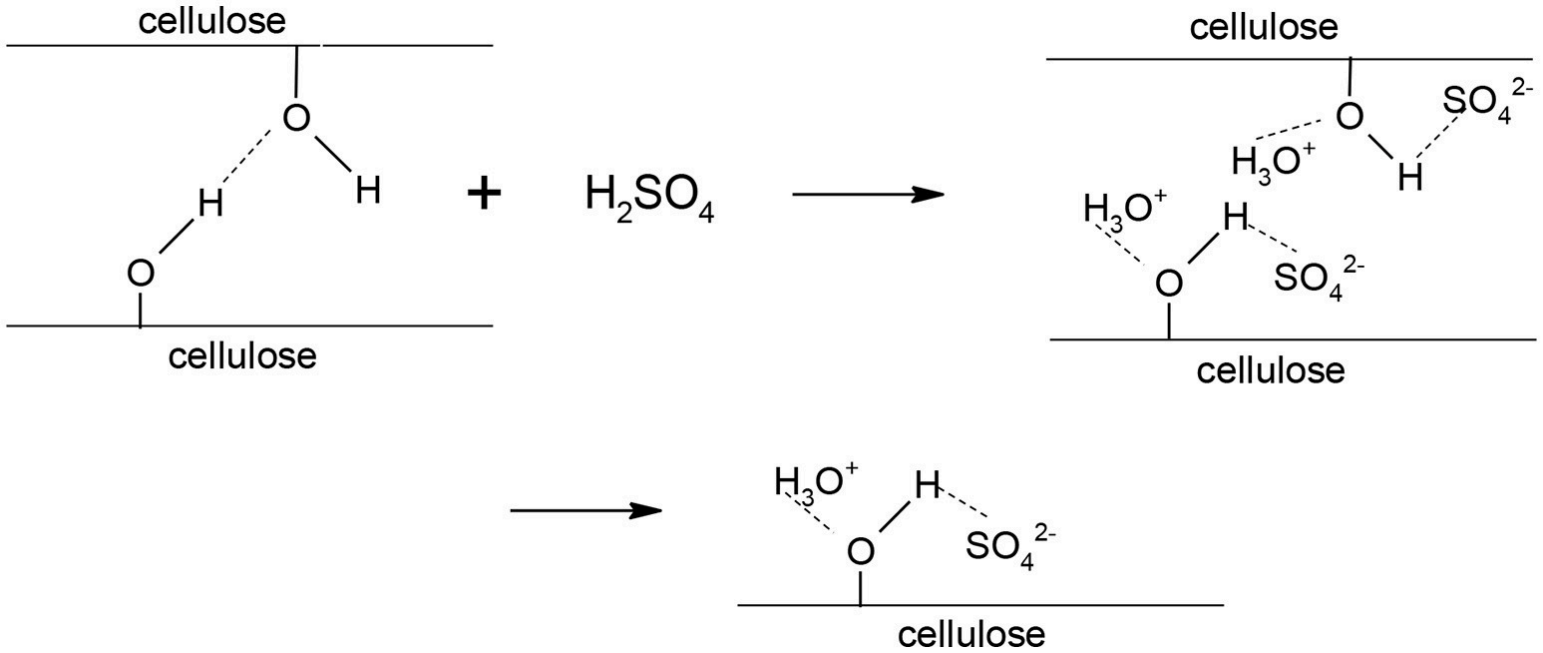
Dissolution mechanism of cellulose in 72 wt% sulfuric acid (based on Feng and Chen, 2008).

This result could be explained by the fact that, at 72 wt% H_2_SO_4_, the molar ratio of H_2_O/H_2_SO_4_ is 2, such that each sulfate anion ends up between two protonated water molecules. Thus, an aqueous solution of sulfuric acid at 72 wt% has some similarities with an ionic liquid since it is a liquid composed exclusively of anions and cations. Ionic liquids have reportedly been used to disrupt the crystalline structure of cellulose before hydrolysis or to simultaneously dissolve and hydrolyze cellulose (Vo et al., 2014), although the mechanism of cellulose dissolution by ionic liquids (and 72 wt% sulfuric acid) is currently not well understood. Feng and Chen (2008) proposed a mechanism for the dissolution of cellulose in an ionic liquid and the latter could readily be applied to the dissolution of cellulose in a 72 wt% sulfuric acid aqueous solution. The sulfate ions and hydronium ions form electron donor-acceptor complexes with the –OH groups in cellulose. As a result of the interactions between cellulose and sulfuric acid, the intra and intermolecular hydrogen bonds between hydroxyl groups in cellulose are broken down, leading to the separation of the molecular chains of cellulose (perceived as swelling) and ultimately cellulose dissolution (Figure 4).

Therefore, the breakdown of the crystalline structure of cellulose is probably one of the most important aspects of the concentrated sulfuric acid pre-treatment. Results also showed that a higher mass ratio of H_2_SO_4_/dry cellulose leads to a higher glucose yield.

#### Influence of pre-treatment time

**Figure 5:**
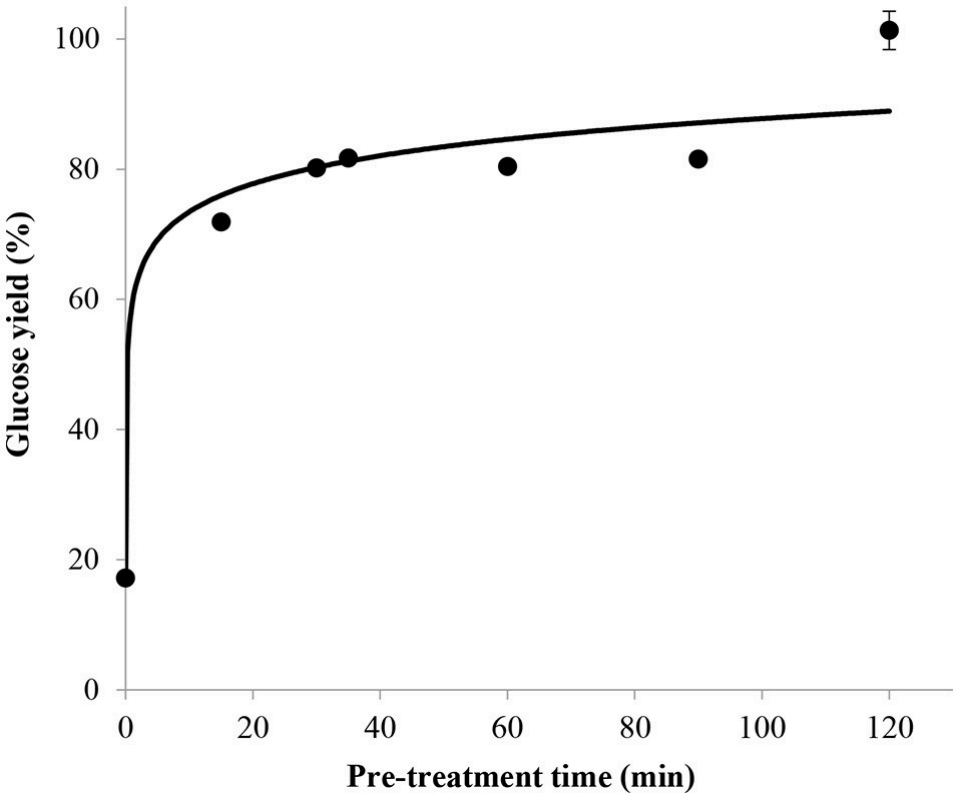
Effect of pre-treatment time on glucose yield with mass ratio of H_2_SO_4_/dry cellulose ratio = 36, 72 wt% sulfuric acid and H^+^/OH^−^ molar ratio = 2.5, combined with a 121°C post-hydrolysis for 10 min.

The glucose yield obtained without pre-treatment in concentrated sulfuric acid before post-hydrolysis was found to be 20% which can mainly be related to the amorphous fraction of cellulose. After only 15 min of pre-treatment in 72 wt% sulfuric acid, the glucose yield rapidly increased to 72% and then continued to increase at a slower rate to reach approximately 100% after 2 h (Figure 5). This long pre-treatment time could be explained by the fact that sufficient time is necessary to convert almost all the crystalline parts into amorphous cellulose, which can then easily be hydrolyzed to glucose during the post-hydrolysis step. Except for the hydrolysate produced after a pre-treatment of 2 h, in which 5-HMF was detected at a concentration below the lower limit of quantification of the HPLC, no 5-HMF peak was observed in the chromatograms obtained from the other pre-treatment times. It was thus deduced that these pre-treatment conditions are not severe enough to cause considerable degradation of glucose, making this process suitable for subsequent fermentation of glucose to ethanol.

According to these results, the most efficient pre-treatment time was 2 h in order to achieve a glucose yield of 100%. Although the influence of pre-treatment time on glucose yield was not investigated for mass ratio of sulfuric acid/dry cellulose of 12 and 24, the best pre-treatment time of 2 h was verified for the most desirable (lowest) ratio of 12 during the other trials determining the influence of concentration of base, post-hydrolysis time and temperature on the glucose yield.

Camacho et al. (1996) studied the formation of glucose by pre-treating microcrystalline cellulose with 70% (w/v) sulfuric acid at a mass ratio of acid/dry cellulose ranging from 1.8 to 9.1, without any post-hydrolysis step. They obtained glucose yields not higher than 35% even if the pre-treatment time was as long as 30 h. Thus, pre-treatment in 72 wt% sulfuric acid is clearly not sufficient to achieve high glucose yields and a post-hydrolysis at reduced acid concentration is necessary to convert amorphous cellulose into glucose. However, adding water to dilute the sulfuric acid from 72 to 4–10 wt% results in a large volume of mixture, which implies higher handling costs. This is why partial neutralization of the sulfuric acid by adding a base was considered as an efficient method to minimize the amount of water to be added for dilution.

### Partial neutralization

#### Influence of acid/base molar ratio

**Figure 6:**
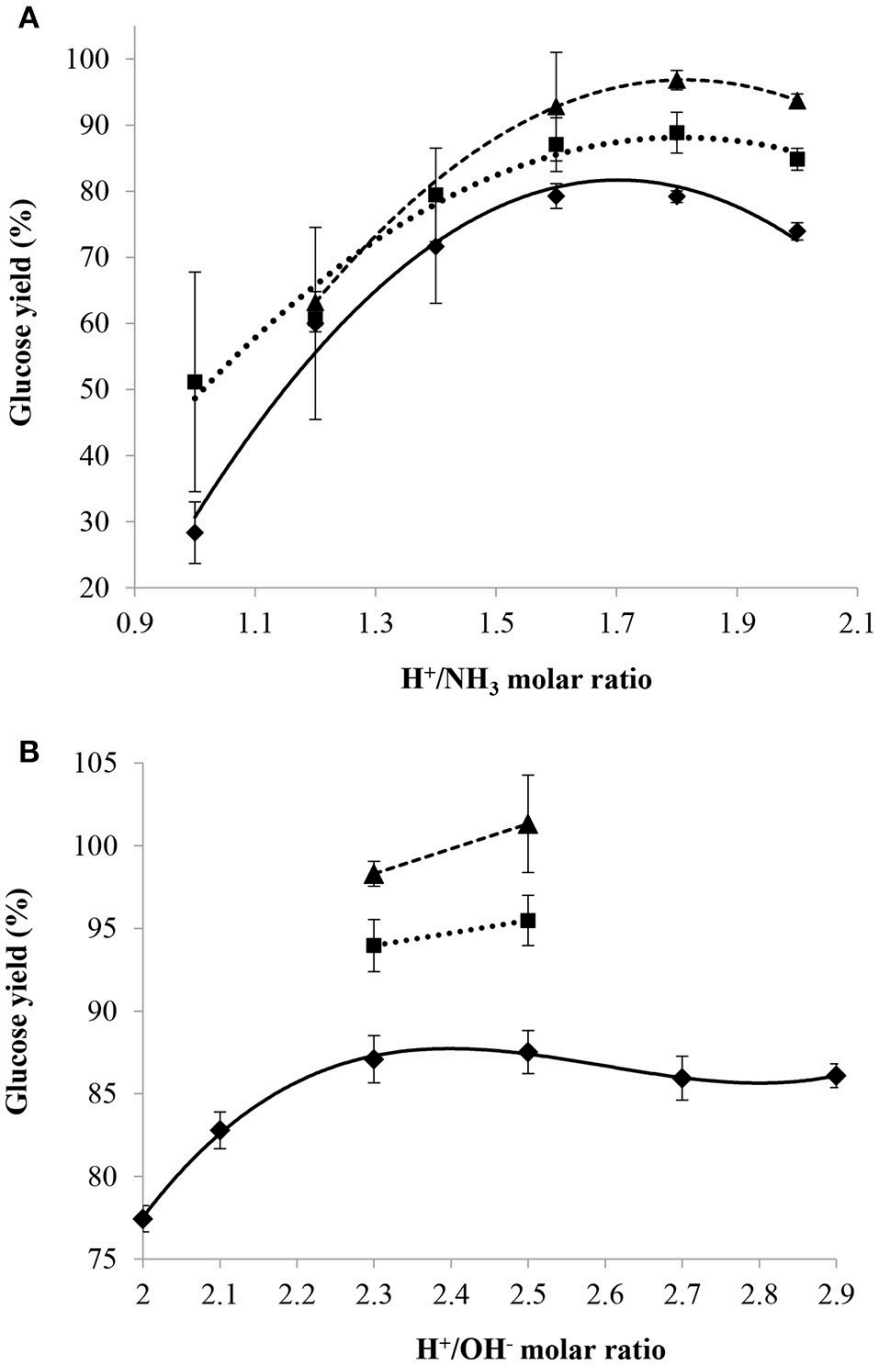
Effect of H^+^/NH_3_ **(A)** or H^+^/OH^−^ (**B**) molar ratio on glucose yield with mass ratio of H_2_SO_4_/dry cellulose ratio = 12(♦), 24(■), 36(▴) combined with a pre-treatment at 30°C and 72 wt% H_2_SO_4_ for 2 h and a post-hydrolysis for 10 min at 121°C.

H^+^/NH_3_ or H^+^/OH^−^ molar ratios were directly used to compare between NH_3(aq)_ and NaOH since both cases involve the neutralization of H_2_SO_4_. Moreover, both ratios were easily calculated because the masses of 95–98 wt% sulfuric acid and 29 wt% aqueous ammonia (or 20 wt% NaOH) added were known. Figure 6 presents the glucose yield for the three values of mass ratio of sulfuric acid/dry cellulose as a function of the H^+^/NH_3_ molar ratio used for partial neutralization with aqueous ammonia (Figure 6A) and as a function of the H^+^/OH^−^ molar ratio used for partial neutralization with sodium hydroxide (Figure 6B).

Poor repeatability was observed for the tests involving H^+^/NH_3_ molar ratios varying from 1.0 to 1.4 which was probably due to the variable evaporation of ammonia during the experiments. This observation was particularly important at low H^+^/NH_3_ molar ratios where the relative amount of NH_3_ compared that of acid is larger than at high H^+^/NH_3_ molar ratios. The glucose yield was 30% when using an H^+^/NH_3_ molar ratio of 1 (low) and a mass ratio of sulfuric acid/dry cellulose of 12. A low H^+^/NH_3_ molar ratio implies that sulfuric acid concentration during post-hydrolysis was low and thus not sufficient to perform an efficient hydrolysis of cellulose into glucose. With higher H^+^/NH_3_ molar ratios, the sulfuric acid concentration during post-hydrolysis was higher and the lower glucose yields observed could be due to glucose degradation, although the concentration of 5-HMF in the hydrolysate was below the lower limit of quantification of the HPLC at these H^+^/NH_3_ molar ratios. Under the conditions tested in this work, the most efficient H^+^/NH_3_ molar ratios were found to range between 1.6 and 1.8.

With regards to using sodium hydroxide to partially neutralize the cellulose and acid mixture before post-hydrolysis, the trials were first conducted with a mass ratio of sulfuric acid/dry cellulose of 12 and, after observing that the highest glucose yield was obtained at a range of H^+^/OH^−^ molar ratio of 2.3–2.5, only this range was investigated for mass ratios of sulfuric acid/dry cellulose of 24 and 36. The most efficient H^+^/OH^−^ molar ratios (2.3–2.5) were subsequently found to be higher than the most efficient H^+^/NH_3_ ratios (1.6–1.8). This observation could be explained by the fact that ammonia is a weak base and thus, more ammonia was needed to produce the required number of moles of hydroxide ions to get the same partial neutralization effect as with sodium hydroxide.

Nevertheless, in both cases, when an H^+^/NH_3_ molar ratio of 1.7 or an H^+^/OH^−^ molar ratio of 2.4 was used, the final mass percentage of sulfuric acid during the post-hydrolysis step was close to 19 wt% although the concentration of the base solutions used for partial neutralization was 29 wt% for ammonia and 20 wt% for sodium hydroxide. Thus, the H^+^/NH_3_ or H^+^/OH^−^ molar ratio providing the highest glucose yield was found to be greatly dependent on the final mass percentage of sulfuric acid used during post-hydrolysis and not related to the type of base used. Despite this observation, the glucose yield under the most efficient conditions was higher with sodium hydroxide than with aqueous ammonia.

#### Influence of NaOH concentration

**Figure 7:**
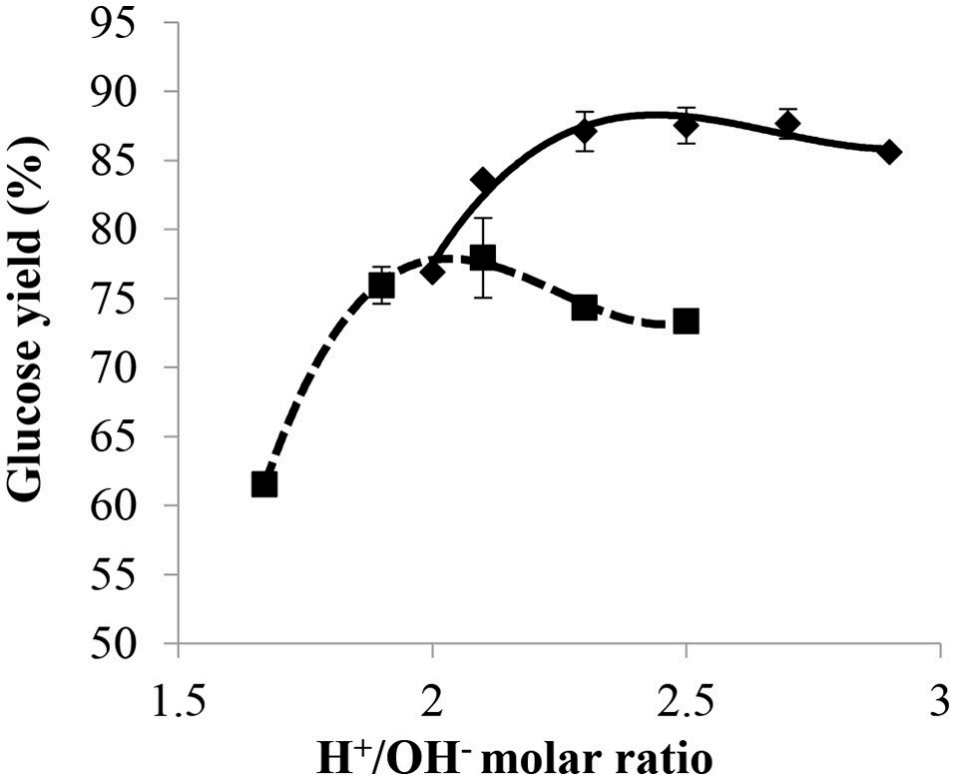
Effect of H^+^/OH^−^ ratio on glucose yield with NaOH concentration of 20 wt% (♦) and 40 wt% (■) with a pre-treatment at 30°C, 72 wt% H_2_SO_4_ for 2 h, mass ratio of H_2_SO_4_/dry cellulose of 12, and post-hydrolysis for 10 min at 121°C.

The effect of NaOH concentration on glucose yield was investigated and the results are given in Figure 7. Solutions of 20 wt% NaOH and 40 wt% NaOH were compared by subjecting the cellulose to a 2 h pre-treatment at 30°C and 72 wt% H_2_SO_4_ for a mass ratio of H_2_SO_4_/dry cellulose of 12, followed by a post-hydrolysis at 121°C for 10 min. The glucose yield vs. H^+^/OH^−^ molar ratio, obtained with both the 20 and 40 wt% NaOH solutions, was found to have the same pattern. Results showed that glucose yield increased with the increasing H^+^/OH^−^ molar ratio until it reached a maximum at the most efficient H^+^/OH^−^ ratio, before gradually decreasing beyond this point. However, the curve obtained with the 20 wt% NaOH solution reached a higher maximum glucose yield at higher H^+^/OH^−^ molar ratios. The most efficient H^+^/OH^−^ ratios with 40 wt% NaOH and 20 wt% NaOH were between 1.9–2.1 and 2.3–2.5, respectively, and the highest glucose yield obtained with the 20 wt% NaOH solution was around 88%, which is about 1.1 times higher than that obtained with 40 wt% NaOH.

With an H^+^/OH^−^ molar ratio of 2 for 40 wt% NaOH and 2.5 for 20 wt% NaOH, the resulting solution after partial neutralization had a mass percentage of sulfuric acid close to 20 wt% in both cases. Thus, the difference in glucose yield observed between the 20 and 40 wt% NaOH solutions used for partial neutralization was most probably due to the quantity of water added with the base during the post-hydrolysis step. This difference in terms of water added together with the base also explains the observations reported in section Influence of Acid/Base Molar Ratio, namely glucose yield under most efficient conditions being higher with 20 wt% NaOH than with 29 wt% NH_3_. Thus, to get a high yield of glucose during post-hydrolysis, it is important to take into account not only the mass percentage of acid but also the quantity of water added during the partial neutralization step since the latter was shown to have a strong effect on the efficiency of post-hydrolysis.

### Post-hydrolysis

#### Effect of time and acid concentration

**Figure 8:**
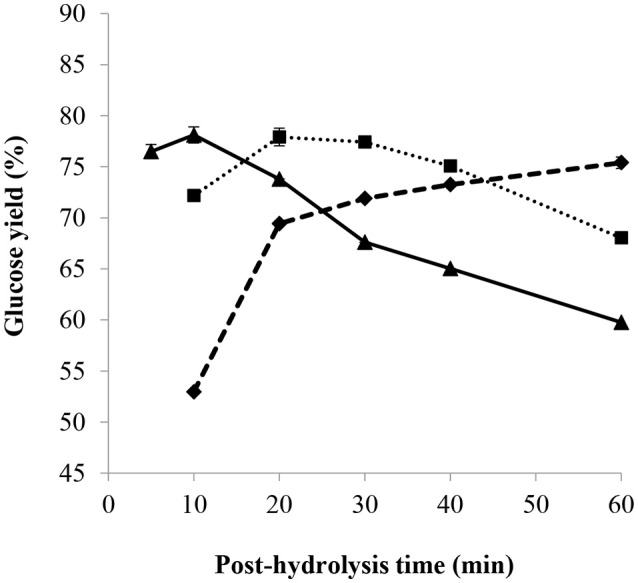
Effect of post-hydrolysis time and H^+^/OH^−^ molar ratio of 1.7(♦), 2.0 (■), 2.3(▴), on glucose yield with a pre-treatment of 2 h at 30°C, 72 wt% H_2_SO_4_ and mass ratio of H_2_SO_4_/dry cellulose of 12, partial neutralization with 32.8 wt% NaOH solution, and post-hydrolysis at 121°C.

The impact of the post-hydrolysis time on the glucose yield using different H^+^/OH^−^ molar ratios is presented in Figure 8. First, it can be observed that the standard deviation among the triplicates at the most efficient conditions (giving highest glucose yields) is very small, such that the associated error bars are hardly distinguishable in Figure 8. With an H^+^/OH^−^ molar ratio of 1.7, increasing the cooking period increased the yield of glucose, whereas with an H^+^/OH^−^ molar ratio of 2.3, increasing the post-hydrolysis time generally led to the opposite scenario. At a low H^+^/OH^−^ molar ratio, the acid concentration in the post-hydrolysis solution was also low such that increasing the cooking time led to the hydrolysis of the remaining amorphous cellulose. However, increasing the post-hydrolysis time at a higher H^+^/OH^−^ ratio probably resulted in the degradation of glucose, although the concentration of 5-HMF in the hydrolysates remained below the lower quantification limit of the HPLC. The most efficient post-hydrolysis time giving the highest glucose yield was found to decrease as sulfuric acid concentration was increased for post-hydrolysis, namely by increasing the H^+^/OH^−^ molar ratio (see Figure 8). This can be of interest in an industrial process since less base would be required for a shorter post-hydrolysis time, producing a glucose-rich hydrolysate. For example, most efficient post-hydrolysis time at 121°C and H^+^/OH^−^ molar ratio of 2.3 was less than 10 min while being 60 min or more for an H^+^/OH^−^ molar ratio of 1.7.

#### Effect of time and temperature

**Figure 9:**
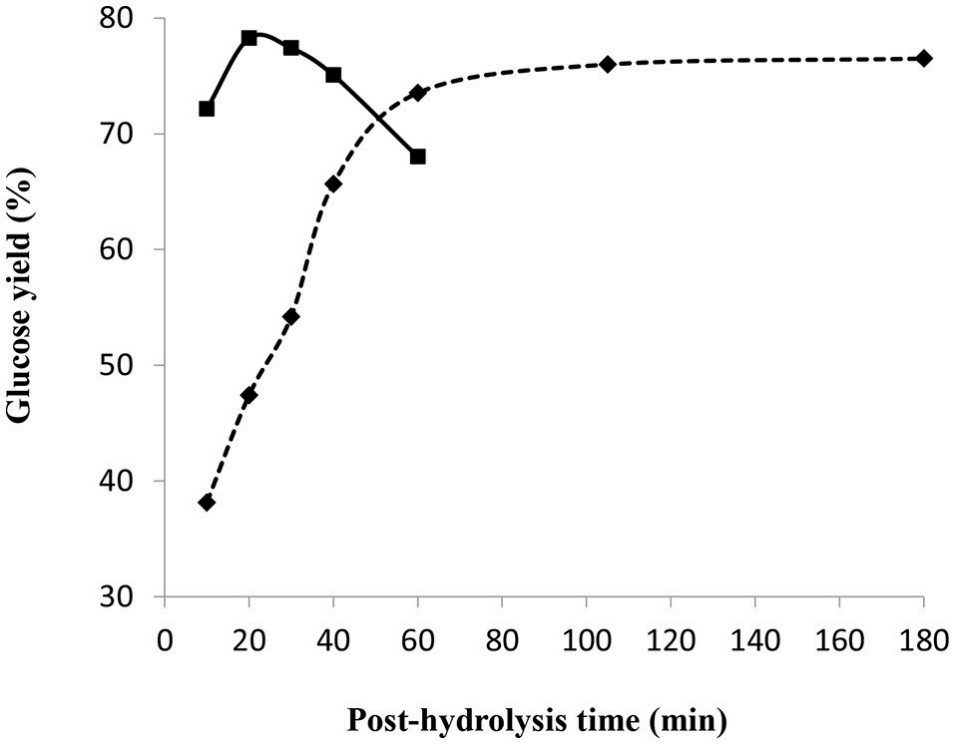
Effect of post-hydrolysis time and temperature of 97°C(♦) and 121°C(■) on glucose yield following a 2 h pre-treatment at 30°C, 72 wt% H_2_SO_4_ and mass ratio of H_2_SO_4_/dry cellulose of 12, followed by a partial neutralization with 32.8 wt% NaOH solution and H^+^/OH^−^ molar ratio of 2.

Figure 9 shows the glucose yield as a function of the post-hydrolysis time at a temperature of 97 and 121°C. Other conditions were kept constant, namely pre-treatment in 72 wt% sulfuric acid for 2 h and mass ratio of acid/dry cellulose of 12 combined with partial neutralization using 32.8 wt% NaOH solution with an H^+^/OH^−^ molar ratio of 2. The standard deviation among the triplicates at the conditions giving highest glucose yield is very small (about 0.4) such that the associated error bars are barely visible in Figure 9. At 97°C, the glucose yield increased with the increasing cooking time to a maximum of around 76%, stabilizing after 60 min. This phenomenon was probably due to the simultaneous occurrence of the saccharification of amorphous cellulose combined with glucose degradation. At 121°C, the glucose yield reached the highest value of 78% (after 20 min), before gradually decreasing to 68% after a total time of 60 min. Glucose degradation seemed to be faster than saccharification at 121°C, as compared to a temperature of 97°C.

Thus, highest glucose yields were obtained at shorter post-hydrolysis times and higher post-hydrolysis temperatures (such as 20 min at 121°C). At a post-hydrolysis temperature of 121°C and with the same conditions as in Figure 9, except for partial neutralization with H^+^/OH^−^ molar ratio of 2.3 and 20 wt% NaOH, the highest glucose yield of 88% was obtained (See Figure 7) for a post-hydrolysis time of 10 min.

The specific impact of using 32.8 wt% NaOH solution for partial neutralization was deduced by considering the glucose yield obtained after a 2 h pre-treatment at 30°C, 72 wt% H_2_SO_4_ and mass ratio of H_2_SO_4_/dry cellulose of 12, partial neutralization with an H^+^/OH^−^ molar ratio of 2 and post-hydrolysis at 121°C for 10 min. As observed from Figures 8, 9, a glucose yield of 72% was obtained with 32.8 wt% NaOH solution while a higher glucose yield of 77% was obtained using 20 wt% NaOH solution for partial neutralization (Figure 7). This confirmed what was noted in section Influence of NaOH Concentration: the amount of water added during the partial neutralization step strongly influences the efficiency of post-hydrolysis.

In their two-step hydrolysis process, Yoon et al. (2014) pre-treated biomass or cellulose with a mass ratio of acid/dry feedstock of 19 at 30°C using an aqueous mixture of 72 wt% H_2_SO_4_ for 1 h, followed by a post-hydrolysis at 4 wt% H_2_SO_4_. The highest cellulose-to-glucose conversion was around 90% for a post-hydrolysis time of 90 min and temperature of 100°C. It was also reported that, regardless of the post-hydrolysis time and temperature, a higher conversion was obtained when hydrolyzing pure cellulose as compared to hydrolyzing a mixture of cellulose and hemicelluloses or a mixture of cellulose, hemicelluloses and lignin, thus justifying the importance of first fractionating biomass and separating it into its constituents.

### Most efficient experimental conditions for cellulose hydrolysis

For post-hydrolysis at 121°C for 10 min using 20 wt% NaOH for partial neutralization, the tested conditions providing the highest glucose yields were found to be as follows: pre-treatment at 30°C with 72 wt% H_2_SO_4_ for 2 h, mass ratio of H_2_SO_4_/dry cellulose of 36, and an H^+^/OH^−^ molar ratio of 2.3–2.5.

With these same post-hydrolysis conditions but using 29 wt% NH_3(aq)_ for partial neutralization, the corresponding conditions giving highest glucose yields were as follows: pre-treatment at 30°C with 72 wt% H_2_SO_4_ for 2 h, mass ratio of H_2_SO_4_/dry cellulose of 36, and an H^+^/NH_3_ molar ratio of 1.6–1.8.

The high yields (close to 100%) obtained with the above best sets of conditions show that this process could nearly be as efficient as the standard ASTM E1758-01 method. Moreover, this hydrolysis process is attractive from an industrial point of view since it can use cellulose as obtained from the fractionation method (60–65% humid), involves smaller volumes, and produces hydrolysates with a higher glucose concentration.

Iranmahboob et al. (2002) used a similar two-step hydrolysis process to produce glucose from dried, ground, and mixed wood chips (50 wt% hardwood and 50 wt% softwood). Their pre-treatment step was done at a mass ratio of H_2_SO_4_/dry wood chips of 2.5 and 80 wt% H_2_SO_4_, and distilled boiling water was added to the so-formed paste to achieve 26 wt% H_2_SO_4_. After stirring the mixture for 30 min at 100°C and filtering it, the filtrate was heated for an additional 2 h. These conditions were reported to be optimum and resulted in an overall conversion efficiency of 78–82% based on theoretical values. Although their process used less acid for the pre-treatment, it required dried feedstock and a much longer post-hydrolysis time than the process used in the present work.

## Conclusion

The individual effect of seven key parameters on the glucose yield of a two-step cellulose hydrolysis process with partial neutralization was studied in this work and the obtained results enabled identifying the conditions giving the highest glucose yield. In the most efficient case, a glucose yield reaching 100% could be achieved and this involved a “swelling” pre-treatment performed at 30°C for 2 h, 72 wt% H_2_SO_4_ and using a mass ratio of H_2_SO_4_/dry cellulose of 36; followed by partial neutralization with 20 wt% NaOH, and an H^+^/OH^−^ molar ratio of 2.3–2.5; and post-hydrolysis at 121°C for 10 min. For the partial neutralization step, sodium hydroxide was the preferred base for higher glucose yields after post-hydrolysis but aqueous ammonia also gave a very high glucose yield (around 95%).

## Author contributions

JK-WC: generated the results by analyzing and interpreting the acquired data, and wrote, drafted and revised the article; XD: produced and checked the results and reviewed the article; VB: performed the experiments and acquired the data; HZ-N: supervised the work by providing an industrial orientation and offered technical guidelines; J-ML: managed and supervised the research, provided scientific and technical guidance, checked the results, and reviewed significantly and approved the article.

### Conflict of interest statement

VB and HZ-N were employed by the company CRB Innovations Inc. The other authors declare that the research was conducted in the absence of any commercial or financial relationships that could be construed as a potential conflict of interest.
